# Community-Based Research as an Alternative to Traditional Research Courses as a Method of Promoting Undergraduate Publication

**DOI:** 10.3389/fpsyg.2019.01012

**Published:** 2019-05-03

**Authors:** Diane Mello-Goldner

**Affiliations:** Pine Manor College, Chestnut Hill, MA, United States

**Keywords:** teaching-research, community-based research, engagement, publishable, undergraduate, research methods

Engaging undergraduate students in research that leads to publishable research is difficult at institutions of all sizes. At small colleges (those with a total enrollment of under 1,000 undergraduate students), it can be even more challenging. One method of addressing this issue is to engage undergraduate students with external community organizations who can provide the students with a larger and more varied data set than if the students conducted research on their own campus. Community-Based Research (CBR) is an effective alternative to teaching an undergraduate research methods course through the participation of off-campus (and sometimes on-campus) community partners (Mettetal and Bryant, 1996). Collaboration, critical thinking, and creativity on the part of both instructors and students are fostered to produce an engaging yet quality research experience. CBR also can be a successful alternative for engaging undergraduate students in publishable research that would be useful at colleges of all sizes.

CBR provides students with real-world experience of developing research and statistical skills along with other career-essential skills such as collaboration, critical thinking, application of knowledge, and social justice. Community partners and students work together to first identify a research topic. In forming a partnership with an academic institution, community members, faculty, and students work together as a research team during all stages of the project–a more collaborative approach than the process in a more traditionally taught research course. Collectively, the research team frames the research question, operationalizes the variables, weighs research design alternatives, details sampling techniques to be utilized, collects and analyzes data, and disseminates the results of their work. By focusing on community-driven questions and ongoing collaboration, CBR provides maximum community input into each stage of the research process. Not only does CBR result in useful publishable data for the community partner and student researchers, but it provides students with the opportunity to apply their research skills in the “real world,” while also helping them learn how to act as socially responsible community members (Ingman, 2016). Research and statistics courses need not only help students develop quantitative reasoning, critical thinking, or problem-solving skills. When CBR involves external partners that are organizations focusing on social and cultural issues, students will develop skills to help them more effectively address issues in their local and/or global community. Consequently, findings of CBR studies are more likely to yield action-based outcomes and be sensitive to ethical concerns and the needs of diverse communities. CBR also provides these community partners, many of whom are non-profit organizations, with the assistance in collecting the data to evaluate their programs needed for continued and future funding opportunities.

At Pine Manor College (a “very” small college with a current total enrollment of under 500 undergraduate students in Chestnut Hill, MA), the Psychology program has been utilizing CBR for more than 10 years. Following this model, engaging in CBR and other types of community partnerships can provide opportunities for colleges of all sizes to have access to large data sets and to provide students with the opportunity for hands-on applied research in a non-academic setting. Given its inclusive nature, CBR holds tremendous promise for all involved by bridging the gap between scholarship and community needs. As collaborators in the research process, the community partners view the college as an ally rather than an esoteric institution that does not have relevance to their mission. Students enrolled in a research methods course that offers a CBR focus are provided with a powerful service-learning opportunity to apply classroom instruction to real-world problem solving while also developing the research skills that will be useful later in graduate school or in their careers. By drawing on the expertise of community members, students develop critical thinking skills while simultaneously gaining sensitivity to community needs (Chapdelaine and Chapman, 1999). The benefits of CBR also extend to other types of partnerships with these community organizations beyond the collaborative research experience for undergraduate students, including networking opportunities for future research opportunities, future internships, or post-graduate job offers (all of which have occurred for the students at Pine Manor College–“When I found my internship it was at Sportsmen's Tennis and Enrichment Center (STEC). It was convenient because I did research there for my Research and Statistics class,” Psychology “17 senior portfolio quote”).

## Process and Outcomes

The CBR experience at Pine Manor College involves a two-semester course sequence across the fall and spring semesters required of all Psychology, Community Health, and Sociology and Political Science majors. Throughout the year students learn about the typical components of the scientific method. The fall semester involves more of an introduction into the various research designs, data collection techniques, and descriptive statistics, with the major paper at the end of the fall semester being a research proposal. During the spring students learn more advanced statistics, and throughout the semester the student groups work with their community partners to refine their project, collect data, analyze the data, and report the data in various forms. A full research report and presentation are the end products of the spring semester. Part of the presentation includes an executive summary shared with the community partner at the end of the semester. Since the purpose of CBR is to create research that can result in positive social change for the community (Strand et al., 2003), students learn how to present results for different audiences. Students also complete a conference-style poster they present to the college community and later submit to a regional psychological association conference (since 2012, typically 100% of students in the CBR class presented at a regional psychology conference with the exception being the 2 years when someone in the Psychology department did not teach the course). By having a college work with the same community partner over several years, this model also can provide students with access to a larger database that could yield more robust findings submitted for publication not only at a regional conference but to potential journals. Below lists examples of the Pine Manor College CBR community partners over the past several years:

- Boys and Girls Club (Boston, MA)
- Transition House-Dating Violence Intervention Program (Cambridge, MA)
- Sportsmen's Tennis and Enrichment Center (Dorchester, MA)
- Dearborn Middle School (Roxbury, MA)
- Partners Hospice (Waltham, MA)
- Cape Verdean Mentoring Program (Roxbury, MA)
- Germaine Lawrence School – a residential treatment center for adolescent girls (Arlington, MA)
- Institute for Community Health (Cambridge, MA)
- The Second Step (Newton, MA)
- The Pine Manor Child Study Center (Chestnut Hill, MA)
- Brookview House–an organization that provides homeless services for women and children in Boston (Dorchester, MA)
- Town of Brookline's Office of Community, Diversity, and Inclusion (Brookline, MA)
- Steps to Success–tutoring program (Brookline, MA).

## Lessons Students Learn From This Experience

The CBR model can be a positive and thought-provoking experience for the students and instructors providing both with insights and interactions with individuals they may not otherwise be aware of or interact with (“…this is another program that I would be really interested to work…” Psychology “18 senior portfolio quote”). There are many benefits to the CBR model for undergraduate students with the most important being that students develop the same research and statistical skills contained in more traditional courses and collect publishable data. Besides the benefits already discussed, some others include the following:

- Students present their projects at a variety of forum and to a variety of groups–the college community, community partner, regional psychology conference. By presenting to community partners (many being non-profit organizations), students learn to communicate science to individuals and groups who have committed themselves to organizations and missions that science could but does not often inform.
- Sometimes, the research topic can be related to a social issue that is personally relevant to the students, “…(the topic of the project)… was all something everyone in the group experienced,” Psychology “18 senior portfolio quote.”
- Students learn the important skill of how to coordinate student and community partner schedules and learn to communicate through various methods. Setting the expectations before the start of the project is essential to prevent miscommunication becoming an issue, and all can learn to function as one team, “Good teamwork is vital for research projects and working with community partners,” Psychology “15 senior portfolio quote.” To ensure effective communication from the start, when soliciting partners, it is a good idea to present them with a description of your program and its goals along with expectations of the partner and questions for the partner to consider before their agreement. Setting a timeline for check-ins between the partner and group/faculty sponsor is also an important element for success. If the partner sees themselves as co-educators in this process, they are more likely to invest in the process and provide helpful feedback to the students throughout the project.
- Students learn about the importance of confidentiality and privacy issues with the collection of data. If the community partner is a non-profit organization that works with a clinical population, undergraduate students may not be able to collect the data themselves. Effective communication and support from the community partner will alleviate this issue as many community partners typically collect data within their organization, “…we also learned about confidentiality when receiving information that is disclosed from others…it's important that you keep it that way,” Psychology “15 senior portfolio quote.”
- Students engage in self-reflection related to the research process as well as to the topic researched, “We needed to analyze our own assumptions as well as the assumptions of our participants when organizing the survey questions and study. Understanding that we could not include certain questions because of the younger participants in the group was just as important to our research as making sure we did include more difficult questions for teens… we respected the perspectives of our partners and the participants to show the other side,” Psychology “16 senior portfolio quote.”
- Along with research and statistical skills, students develop a sense of civic engagement and social justice that may not be possible in more traditional “shelf-research” undergraduate projects. This may influence the students to pursue future work with the same community partner, “We demonstrated social responsibility by participating in an afterschool program research for the younger generation to promote their interest in reading and education. I particularly got very involved and decided that I wanted to become an intern at the program,” Psychology “12 senior portfolio quote.”
- Evaluation is critical to ensure that CBR is successful for the students and partners. In the Pine Manor College CBR courses, the research process and group members are evaluated by the instructor (through their paper and group participation grade), as well as by peers, and the community partner. Community partners are also asked to reflect upon their experience at the end of the process to highlight what they perceived as successes as well as limitations and challenges.

## Conclusion

Engaging undergraduate students in the research and publication process is challenging at any college. At colleges that do not have large research participant pools or active faculty research programs, the challenge is even greater. CBR presents an effective and engaging alternative to teaching research methods and provides students with the opportunity to conduct a research project that can result in a meaningful presentation and publication. At Pine Manor College, when seniors reflect upon their CBR experience in their senior portfolios (a graduation requirement that requires students to reflect upon the College's learning outcomes), almost all Psychology majors (92% over the last 2 years) mention how their CBR project allowed them to demonstrate their critical thinking skills, effective communication (written, visual, and oral), collaboration skills, citizenship and social responsibility, and application of knowledge. Students also describe how the experience helped them to develop a deeper understanding of their own cultural and global self-awareness, which will assist them in being more effective in their various future roles: “Being in those environments drew me out my comfort zone because it challenged me to find ways to improve these adolescent's skills,” Psychology “17 senior portfolio quote.” An end of semester survey assessing students' perceptions of the class found that students felt that CBR was a better way to learn about the concepts of research methods and statistics because it was a more active approach (*M* = 4.62 on a 5-point scale with higher numbers indicating more positive ratings). The benefits to this method of teaching research far outweigh the costs, as students are engaged in publishable research while also developing important skills that are especially important for those who wish to work in the psychology field.

## Author Contributions

DM-G wrote the manuscript based on past experiences.

### Conflict of Interest Statement

The author declares that the research was conducted in the absence of any commercial or financial relationships that could be construed as a potential conflict of interest.
